# Positive Impacts of a Four-Week Neuro-Meditation Program on Cognitive Function in Post-Acute Sequelae of COVID-19 Patients: A Randomized Controlled Trial

**DOI:** 10.3390/ijerph20021361

**Published:** 2023-01-11

**Authors:** Christophe Hausswirth, Cyril Schmit, Yann Rougier, Alexandre Coste

**Affiliations:** 1 LAMHESS, Université Côte d’Azur, EA6312, 06205 Nice, France; 2 BeScored Institute, Valbonne Sophia Antipolis, 06560 Valbonne, France; 3 Faculty of Health, University of Technology (UTS), Sydney, NSW 2007, Australia; 4 WHealth Found, 06270 Villeneuve-Loubet, France

**Keywords:** post-acute COVID-19 syndrome, mindfulness, mental health, cognitive tasks

## Abstract

Study objective: Long COVID patients can experience high levels of impairment in their cognitive function and mental health. Using a parallel randomized control trial, we evaluated the effectiveness of a neuro-meditation program to reduce cognitive impairment in patients with long COVID. Methods: A total of 34 patients with long COVID were randomized to an intervention group (G-Int; *n* = 17) or a control group (G-Con; *n* = 17) and 15 healthy participants were constitutive of a normative group (G-Nor). The intervention consisted of ten 30-min sessions of Rebalance^®^ over a period of five weeks. Each session included sound therapy and coach-guided meditation associated with light stimulations (i.e., chromotherapy). Primary outcomes were performance on five computerized cognitive tasks (*choice response time*, *pattern comparison*, *Simon, pursuit rotor task*, *and Corsi block-tapping task*), mental and physical fatigue (*Chalder fatigue scale*), perceived stress (*perceived stress scale*) and mood (*profiles of mood states*). Secondary outcomes were anxiety and depressive symptoms (*hospital anxiety and depression scale*), muscular pain, joint pain, and headaches using visual analog scales (*VAS*) as well as sleep quality (*Spiegel sleep questionnaire*). Assessments were conducted at baseline and at 1–2 and 7–8 days of follow-up. Results: Compared to healthy subjects, long COVID patients showed significant differences at baseline on all the self-report questionnaires, and a Rebalance^®^ program improved all the subjective reports, as well as cognitive performances, especially on reaction time-based tasks. In particular, only the G-Int group revealed shortened reaction times in the choice reaction time (RT_baseline_ = 593 ± 121 ms vs. RT_post2_ = 521 ± 86 ms, *p* < 0.001), Simon (RT_baseline_ = 539 ± 123 ms vs. RT_post2_ = 494 ± 134 ms, *p* < 0.01), and pattern comparison tasks (RT_baseline_ = 1244 ± 315 ms vs. RT_post2_ = 1079 ± 213 ms, *p* < 0.001). Conclusions) Initial evidence suggests that neuro-meditation reduces cognitive impairment and improves physical and mental fatigue, muscle and joint pain, symptoms of depression and anxiety, mood disturbances as well as sleep quality. The Rebalance^®^ program hence constitutes a promising non-pharmacological intervention for the treatment of long-term psychological/cognitive outcomes of COVID-19.

## 1. Introduction

The devastating health consequences of acute COVID-19 infection have been thoroughly characterized, and growing concerns regarding potential long-term incidences have arisen for patients who survive the infection and may experience long-term effects. Indeed, many survivors have revealed chronic and debilitating symptoms long after viral recovery, despite mild illness severity at onset [1], pointing to residual effects within the peripheral and central nervous system (CNS). Long COVID is characterized by persistent symptoms that are still present at least 4 weeks after initial infection, and often last for several months [2]. In this context, scientific and medical communities have identified an urgent need to investigate this syndrome, known as post-acute sequelae of COVID-19 (PASC; National Institutes of Health, 2021), in order to develop effective therapies and to prevent further escalation of adverse health outcomes.

Few studies have documented the most common persistent symptoms after severe COVID-19 infection. These symptoms include fatigue, dyspnea, “brain fog”/various cognitive symptoms, pain, anxiety, depression, and gastrointestinal issues [2,3,4]. In these cohorts, the symptoms arising from COVID-19 increased disability and negatively impacted physical function and quality of life [1] and affected participation in general life activities and the ability to work [5]. There are common reports of “brain fog” with self-reported broad psychological symptoms including low energy, concentration problems, disorientation, and difficulty finding the right words. In parallel, case studies have provided evidence that COVID-19 patients can develop a range of neurological complications [6] including those arising from strokes [7], encephalopathies [8], inflammatory syndromes and microbleeds [7], and autoimmune responses [9]. Then, there are concerns regarding general cognitive performance with long COVID patients.

Interventions that support mental health and psychological well-being have been shown to help people with physical health challenges [10]. For instance, mindfulness-based interventions (MBI) are known to strengthen resiliency in vulnerable populations, then encouraging disease management for an improved quality of life [11]. More recently, it has also been shown that neuro-meditation reduces excessive sympathetic activity, promoting enhanced sleep quality and autonomic control during periods of increased work-related stress, for instance for nursing staff and surgeons [12]. It is therefore important to consider such interventions within integrated models of care for long COVID. However, since long COVID is an emerging clinical entity, there remains a critical gap in the literature regarding effective interventions for this complex condition.

The purpose of the present study was thus to evaluate the influence of an MBI program on cognitive performance in long COVID patients. We hypothesized that a four-week MBI using neuro-meditation would improve the overall cognitive functioning of all participants and reduce states of mental and physical fatigue, and confusion.

## 2. Materials and Methods

### 2.1. Participants

Thirty-four patients with long COVID and fifteen healthy participants were recruited for this study (see Table 1). Patients were randomly allocated to an intervention (G-Int; *n* = 17) or control (G-Con; *n* = 17) group while healthy subjects were constitutive of a healthy control group (i.e., normative group, G-Nor; *n* = 15). Inclusion criteria for the intervention and the control group were an age of 18 and older, the absence of dementia or neuropsychiatric disorders, a diagnosis of long COVID confirmed by SARS-CoV-2 reverse transcription-polymerase chain reaction (RT-PCR) or serology and scores in screening questionnaires ≥8 for the *Hospital Anxiety and Depression Scale* (*HADS-A* and/or *HADS-D* subscales; [13]), ≥4 for the *Chalder Fatigue Scale* (global binary fatigue score; [14]) and ≤17 for the *Spiegel Sleep Quality* questionnaire (*SSQ*; [15]). Inclusion criteria for the healthy normative group were an age of 18 and older, the absence of dementia or neuropsychiatric disorders, as well as the absence of SARS-CoV-2 virus infection confirmed by a serological test and the same screening questionnaires mentioned above with scores on *HADS-A* and *HADS-D* < 8, global binary fatigue score (*CFQ 11*) < 4 and *SSQ* > 17. The study doctor was in contact with the hospitals to ensure that participants were diagnosed with long COVID before inclusion in our study.

### 2.2. Study Design and Procedures

**Figure 1:**
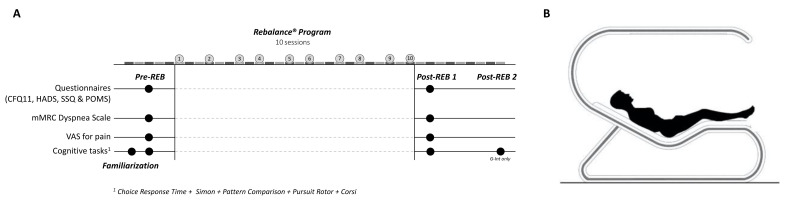
(**A**) Experimental design. (**B**) Schematic representation of the Rebalance^®^.

A parallel randomized control trial was used to establish the effectiveness of an MBI using the Rebalance^®^ technology (Rebalance Tech Corp., Miami, FL, USA). Figure 1A provides an overview of the experimental design and measurements as well as a schematic representation of the technology used (Figure 1B).

The G-Int group members followed a Rebalance^®^ Program while the G-Con and G-Nor groups did not receive any treatment. Before and after the intervention period, baseline and post-intervention measurements, including various cognitive tasks and self-administered questionnaires were obtained to determine the changes made by the Rebalance^®^ Program. Because it has been shown that the Rebalance^®^ program can have delayed effects [16], a second post-assessment was carried out one week after the first session for the G-Int only.

#### 2.2.1. The Rebalance Program

The Rebalance program included a total of ten 30-min sessions spread over four weeks (i.e., two to three sessions a week). Each session of neuro-meditation was performed by means of the Rebalance^®^ device. Rebalance^®^ is a non-invasive cognitive stimulation and mindfulness training device based on the latest advances in the applied neuroscience [11,16]. During the sessions, the participants lay down in a “zero gravity” position (Figure 1B). The 30-min mindfulness training included sound therapy and coach-guided meditation associated with light stimulations (synchromotherapy^®^). The software adapted the frequency of light stimulations based on the real-time reading of the dominant wave of the participant’s brain at the beginning of the Rebalance session using a 4-channel EEG system (headband Muse 2 InteraXon Inc., Toronto, ON, Canada; [17]). More specifically, five frequency levels were used in real-time, allowing for an observed dominant wave to evolve gradually (3–4 min) toward the target brain biorhythm, namely, the dominant range theta (between 4 and 7 Hz) and the alpha waves (between 8 and 13 Hz). As such, the sessions should be seen as interactive due to the reciprocal influences between the participant’s adjustments to the device instructions (e.g., attentional focus or body movements) and the device adjustments to the participant’s brain activity (e.g., type and frequency of the stimulation). This device has already been the subject of several scientific studies (e.g., [12,16]) and has been previously well described (see [12]).

#### 2.2.2. Measures

##### Self-Administered Questionnaires

All participants completed questionnaires assessing their sleep quality, mood, anxiety and depression states, perceived physical and mental fatigue, the severity of dyspnea, as well as levels of muscle and joint pain and headaches. Questionnaires were administered in the days preceding the first MBI session (i.e., pre-REB session) and 24 h after the last session (i.e., post-REB session) and included the *Spiegel sleep quality* (*SSQ*, 6 items, 6 choices per item; [15,18]), the *abbreviated profile of mood states* (*POMS*, 40 items, 5 choices per item, 7 subscales; [19]), the *hospital anxiety and depression scale* (*HADS*, 2 subscales, 7 items per subscale, 4 choices per item; [13]), the *Chalder fatigue scale* (*CFQ 11*, 11 items, 4 choices per item, 2 subscales of 7 and 4 items, respectively, for physical fatigue and mental fatigue; [14]), the *modified Medical Research Council dyspnea scale* (*mMRC*, 5 grades ranging from 0 to 4; [20]) as well as visual analog scales for pain (headaches, muscle, and joint pain, 100 mm VAS [21]).

##### Cognitive Tasks

In addition to the questionnaires, participants performed five computerized cognitive tasks selected from the open-source software “*The Psychology Experiment Building Language*” (*PEBL*, version 2.1, 2019), twice and in a randomized manner to prevent any potential order effect. Furthermore, a familiarization phase including all five cognitive tasks was carried out two days prior to initial testing to prevent any potential learning effect. Practice trials (≃one minute on each task) were also realized before starting recording in each evaluation session.

1. Choice Response Time (CRT)

The choice response time task (Figure 2.1; [22]) consists of the visual detection of a letter (a, d, c, e, o, or n) displayed very briefly (100 ms) at the center of a screen (24 inches). After detection, the participant must select this letter as fast as possible among a choice of two letters displayed on the right and the left side of the screen using two push buttons (*CompuPhase*, Bussum, The Netherlands) held in each hand. In total, the participants performed two blocks of ninety trials (≃three minutes each). Stimulus onset asynchrony (i.e., the time that elapses between two successive stimuli) and timeout (maximum time to respond before automatically switching to the next trial) were set to 2000 and 1500 ms, respectively.

2. Pattern Comparison Task (PC)

The pattern comparison task is a visual–spatial discrimination task (Figure 2.2; [23]) in which the participant has to indicate as quickly and accurately as possible whether two 4 × 4 grid patterns of circles presented simultaneously side by side are identical or different by pressing push buttons held in their right and left hand. Accordingly, CO trials correspond to identical patterns while IN trials include different grid patterns. In total, the participants performed two blocks of ninety-six trials (≃three minutes each), including half CO trials and a half IN trials.

3. Simon Task

The Simon task is a selective inhibition task in which the participant is required to respond as quickly and accurately as possible to the color (blue or red) of a stimulus, irrespective of its location on the right or left side of the screen (Figure 2.3; [24]). Accordingly, the task includes two types of trials: congruent trials (CO, both the stimulus location and color induce the same response) and incongruent trials (IN, the stimulus location and color elicit opposite responses). During IN trials, participants had to overcome the irrelevant dimension of the stimulus in order to give the correct response, then probe the efficiency of selective response inhibition. Each trial began with the presentation of a cross at the center of the screen as a fixation point. After 400 ms, the stimulus was presented, and participants had to respond according to the color of a circle (50 pixels in diameter) located either on the right or left side of the screen (300 pixels apart from the middle), using two push buttons held in each hand. The delivery of the response turned off the stimulus. In total, the participants performed two blocks of one hundred trials (≃three minutes each), including half CO trials and a half IN trials.

4. Pursuit Rotor Task

Fine motor skills and visuospatial coordination abilities were measured using the pursuit rotor task (Figure 2.4; [25]). During this task, the participants are required to track, using a mouse cursor, a target (red circle of 50 pixels in diameter) that moves steadily along a circular path (radius = 253 pixels). The rotation speed was one revolution every 7.5 s i.e., four rotations for a 30 s trial. In total, two blocks of five trials were carried out in each evaluation session.

5. Corsi Block-Tapping Task

The Corsi block-tapping task is a visuospatial short-term working memory task (Figure 2.5; [26,27]) in which the participant has to reproduce immediately, after its presentation on a screen, a sequence of visual patterns (blue square that lights up successively in yellow) using the computer mouse. The number of elements to be memorized increases progressively (starting at three up to nine elements) by increments of two trials of the same length until two consecutive failures, marking the end of the test. The duration of the task then depends on the subject’s performance. The task was performed twice in each evaluation session.

#### 2.2.3. Data Analysis

All data were stored in an electronic database, then preprocessed and analyzed using Matlab R2014b (*The MathWorks*, Natick, MA, USA) and the open-source JAMOVI software (version 0.8.1.7, Sydney, Australia). For cognitive task results, all dependent variables were averaged over the two experimental rounds. For cognitive tasks 1–3, the dependent variables were the response accuracy (i.e., the percentage of correct responses) and response times (RT in ms), whereas the dependent variables for tasks 4 and 5 were:

- The percentage of time-on-target and the mean deviation of the mouse cursor from the target (in pixels) for the pursuit rotor task;
- The block span (i.e., the longest length at which at least one pattern was correctly recalled) and the total score (i.e., the span × the number of trials total that were correctly recalled) for the Corsi block-tapping task.

Note that for cognitive tasks 1–3, the ten first trials on each testing session were removed from the analysis. Similarly, RTs of less than 100 ms and RTs higher than 1500 ms for the Simon task [28] or higher than 2000 ms for the PC task were considered anticipated responses and omissions, respectively, and were excluded from the analysis.

Repeated-measure ANOVAs, and, when needed, Tukey (HSD) post hoc tests, were then used to determine whether there was a statistically significant difference between the means of the three groups (G-Nor, G-Con, and G-Int) on the different dependent variables. The three groups were considered as a between-subjects factor (group), whereas the time of the evaluation (pre, post) was considered as the within-subject factor (time). The sphericity was assessed using Mauchly’s test, and a Greenhouse–Geisser (G-G) correction was used when sphericity could not be assumed. All results were presented as mean ± standard deviation (SD) and statistical significance was accepted at *p* < 0.05. Effect sizes of the repeated measures ANOVAs are reported in Section 3 using partial eta squared η_p_^2^ [29] and interpreted according to Cohen’s guideline [30], where 0.02 corresponds to a small effect, 0.13 to a medium effect, and 0.26 to a large effect. 

## 3. Results

### 3.1. Self-Report Data

The mean scores ± standard deviation of all questionnaires for the three groups (G-Nor, G-Con, and G-Int) are presented in Table 2. Moreover, internal consistency (Cronbach’s alpha) for the different self-administered questionnaires ranged from acceptable (0.8 > *α* ≥ 0.7) to excellent (*α* ≥ 0.9) as shown in Table 3. A significant time–group interaction (3 *×* 3) was observed in CFQ 11 for both physical fatigue (F(2,46) = 87.2, *p* < 0.001, η_p_^2^ = 0.79) and mental fatigue subscale (F(2,46) = 79.1, *p* < 0.001, η_p_^2^ = 0.77), in HADS for both anxiety (F(2,46) = 3.74, *p* < 0.05, η_p_^2^ = 0.14) and depression subscale (F(2,46) = 22.1, *p* < 0.001, η_p_^2^ = 0.49), in VAS for both muscle and joint pain (F(2,46) = 3.46, *p* < 0.05, η_p_^2^ = 0.13) and headaches (F(2,46) = 5.28, *p* < 0.01, η_p_^2^ = 0.19), in POMS for tension (F(2,46) = 14.1, *p* < 0.001, η_p_^2^ = 0.38), anger/hostility (F(2,46) = 8.16, *p* < 0.001, η_p_^2^ = 0.26), fatigue/inertia (F(2,46) = 13.8, *p* < 0.001, η_p_^2^ = 0.37), depression/dejection (F(2,46) = 5.68, *p* < 0.01, η_p_^2^ = 0.20), esteem-related effect (F(2,46) = 9.06, *p* < 0.001, η_p_^2^ = 0.28), and confusion/bewilderment (F(2,46) = 12, *p* < 0.001, η_p_^2^ = 0.34) subscales, as well as the POMS total mood disturbance scores (F(2,46) = 12.4, *p* < 0.001, η_p_^2^ = 0.35). While we did not find a significant time–group interaction for the POMS vigor subscale (F(2,46) = 1.42, *p* = 0.25, η_p_^2^ = 0.06) or the mMRC (F(2,46) = 2.82, *p* = 0.07, η_p_^2^ = 0.11), the results, however, showed a main effect of group (F(2,46) = 16.4, *p* < 0.001, η_p_^2^ = 0.42) and a main effect of time (F(1,46) = 5.4, *p* < 0.05, η_p_^2^ = 0.1) in mMRC, as well as a main effect of group (F(2,46) = 56.1, *p* < 0.001, η_p_^2^ = 0.71) in the POMS vigor subscale. 

Overall, post hoc pairwise comparisons using Tukey (HSD) tests indicated that patients’ scores (whether G-Int or G-Con) differ significantly from healthy subjects’ scores (see Table 2 for more details and *p*-values). This is true at least for the baseline evaluation. Indeed, while post hoc analyses revealed no differences between Pre-Post testing sessions in G-Con and G-Nor, we observed for G-Int a significant reduction of the score of physical fatigue (−13.0 points, *p* < 0.001), mental fatigue (−7.7 points, *p* < 0.001), anxiety (−3.6 points, *p* < 0.001), depression (−5.2 points, *p* < 0.001), muscle and joint pain (−1.8 points, *p* < 0.01), headaches (−2.5 points, *p* < 0.001), tension (−5.7 points, *p* < 0.001), anger/hostility (−5.4 points, *p* < 0.001), fatigue/inertia (−5.5 points, *p* < 0.001), depression/dejection (−5.2 points, *p* < 0.001), confusion/bewilderment (−5.3 points, *p* < 0.001), and total mood disturbance (−31.3 points, *p* < 0.001), as well as an increase in the score reflecting sleep quality (+3.4 points, *p* < 0.05) and the esteem-related affect (+2.8 points, *p* < 0.01).

**Figure 3:**
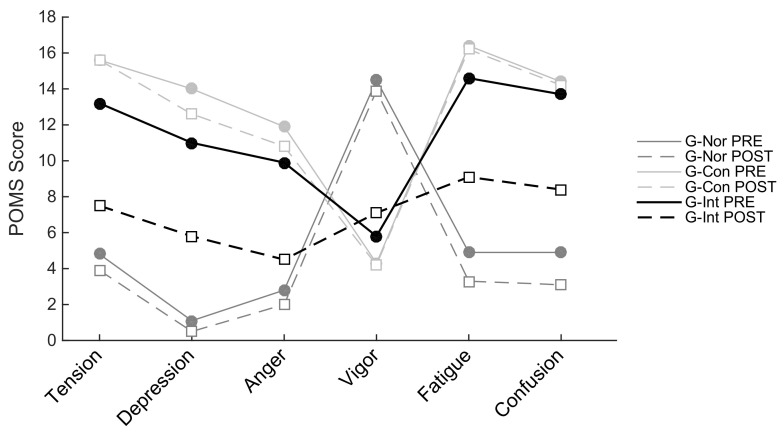
*Profile of mood states* (*POMS*) profiles at two different time points: Before the program (PRE; solid line) and after the program (POST; dashed line) for the healthy (active) group (G-Nor; in dark gray), the control group (G-Con; in light gray), and the intervention group (G-Int; in black).

Figure 3 provides a visual representation of the five negative mood states (tension, depression, anger, fatigue, and confusion) and one positive mood state (vigor) of the *POMS* questionnaire. 

From this, it can be seen that healthy/active people (i.e., G-Nor participants) are characterized by the so-called “iceberg profile” [31], which is lower levels of negative mood states and higher level of positive mood state, while long COVID patients (G-Con and G-Int) are characterized by an “inverted profile” (i.e., a lower level of vigor and higher levels of tension, depression, anger, fatigue, and confusion than the average individual). Remarkably, after 10 sessions of neuro-meditation using the Rebalance^®^ device, the *POMS* profile of the G-Int group tended to become closer to the G-Nor group, whereas no significant change in pre–post sessions was observed for G-Con and G-Nor. 

### 3.2. Cognitive Tasks 

The primary outcome measures for the five cognitive tasks, for G-Nor, G-Con, and G-Int, are reported in Table 4. 

For the choice response time task, the repeated-measure ANOVA performed on RTs revealed a main effect of time factors (G-G corrected: F(1.20,55.34) = 8.67, *p* < 0.01, η_p_^2^ = 0.16) and an interaction between group and time factors (G-G corrected: F(2.41,55.34) = 3.22, *p* < 0.05, η_p_^2^ = 0.12). Post hoc tests indicated that only the G-Int had a significant reduction in RTs (−9.8% between baseline and Post1, *p* < 0.01; −12.1% between baseline and Post2, *p* < 0.001). However, no significant differences between groups (F(2,46) = 1.49, *p* = 0.23, η_p_^2^ = 0.06) or time (G-G corrected: F(1.21,55.86) = 1, *p* = 0.34, η_p_^2^ = 0.02) were found on response accuracy.

For the pattern comparison task, the repeated-measure ANOVA with group as the between-subjects factor and Pre, Post1, and Post2 values as the within-subjects factor conducted on response accuracy of CO trials revealed an interaction among the two factors (F(4,92) = 4.85, *p* < 0.001, η_p_^2^ = 0.17). Post hoc analyses revealed a significant decrease in accuracy between baseline and Post1 for the G-Nor group only (−2.1%, *p* < 0.05). Repeated-measure ANOVA conducted on response times of CO trials revealed a main effect of Group (F(2,46) = 7.16, *p* < 0.01, η_p_^2^ = 0.24), with longer RTs for G-Con (+401 ms, *p* < 0.001) and G-Int (+243 ms, *p* = 0.06) than G-Nor. Regarding IN trials, the results on response accuracy showed only a main effect of Group (F(2,46) = 4.98, *p* < 0.05, η_p_^2^ = 0.18), with higher accuracy for G-Con (+0.02, *p* = 0.07) and G-Int (+0.03, *p* < 0.01) than G-Nor. Interestingly, the results on RTs showed a main effect of Time (F(2,92) = 4.86, *p* < 0.01, η_p_^2^ = 0.10) with faster RTs at Post1 than at baseline (−40.6 ms), a main effect of group (F(2,46) = 4.50, *p* < 0.05, η_p_^2^ = 0.16) with faster RTs for G-Nor (990 ms ±146) than G-Int (1160 ms ± 264) and G-Con (1276 ms ± 378), as well as an interaction between these two factors (F(4,46) = 2.84, *p* < 0.05, η_p_^2^ = 0.11). Post hoc analyses revealed that G-Int showed the highest reduction in RTs between baseline and Post1 (−7.1% vs. −3.2 for G-Nor and −0.2 for G-Con). Moreover, a significant reduction in RTs was observed at Post2 for G-Int (−13.3% between baseline and Post2, *p* < 0.001). 

For the Simon task, the repeated-measure ANOVA performed on the response accuracy of CO trials revealed only a main effect of group (F(2,46) = 4.88, *p* < 0.05, η_p_^2^ = 0.17), with lower accuracy for G-Nor (−0.01, *p* < 0.05) and G-Int (−0.01, *p* < 0.05) than G-Con. A main effect of time (G-G corrected: F(1.71,78.76) = 7.20, *p* < 0.01, η_p_^2^ = 0.14) was found for IN trials’ RT. In particular, a significant reduction in RT between baseline and Post2 sessions was observed for the intervention group only (−8.4%, *p* < 0.01). No other main effect or interaction reached the statistically significant level. 

For the pursuit rotor task, the repeated-measure ANOVA performed on the percentage of time spend on target revealed a main effect of Group (F(2,46) = 3.85, *p* < 0.05, η_p_^2^ = 0.14) as well as an interaction between time and group factors (G-G corrected: F(2.22,51.01) = 3.86, *p* < 0.05, η_p_^2^ = 0.14). Tukey’s post hoc difference tests indicated that G-Con spent greater time on target (+1.4%, *p* < 0.05) than G-Nor during the post session. Regarding the mouse deviation, results showed a main effect of group (F(2,46) = 5.61, *p* < 0.01, η_p_^2^ = 0.20), with higher deviation for G-Con (+3,75 pixels, *p* < 0.01) than G-Nor.

Finally, for the Corsi block-tapping task, results for the total score showed a main effect of time (G-G corrected: F(1.25,57.43) = 9.38, *p* < 0.01, η_p_^2^ = 0.17), a main effect of group (F(2,46) = 12.1, *p* < 0.001, η_p_^2^ = 0.35), and an interaction between these two factors (G-G corrected: F(2.50,57.43) = 15.64, *p* < 0.001, η_p_^2^ = 0.40). Post hoc tests indicated lower scores for G-Con (−17.3, *p* < 0.001) and G-Int (−18.2, *p* < 0.001) than G-Nor, as well as a significant increase in score between the Pre-Post sessions for G-Int only (+22.4%, *p* < 0.001). Repeated-measure ANOVA performed on the length of the longest sequence revealed a main effect of group (F(2,46) = 8.1, *p* < 0.001, η_p_^2^ = 0.26) and a group–time interaction (G-G corrected: F(2.52,57.87) = 2.93, *p* < 0.05, η_p_^2^ = 0.11). Post hoc comparisons indicated a shorter sequence for G-Con (−1.1 items memorize, *p* < 0.01) and G-Int (−1 items memorize, *p* < 0.01) than G-Nor, as well as a tendency to a higher sequence length between the pre–post sessions for the normative group only (+0.4 items memorized, *p* = 0.09). 

## 4. Discussion

This study aimed at testing the effectiveness of an MBI in improving cognitive-related parameters in patients with long COVID after the SARS-CoV2 pandemic. The main findings of this study were that ten 30-min sessions of neuro-meditation using the Rebalance^®^ device can significantly improve mood, physical, and mental fatigue, as well as cognitive functioning in people living with long-term effects of COVID-19.

Regarding the effect of the intervention on the subjective reports, the findings were largely in line with our hypothesis. Patients receiving MBI showed significant decreases in physical and mental fatigue, muscle and joint pain, symptoms of depression and anxiety, and mood disturbances, and had significantly better improvements in sleep quality (increasing significantly by 3.4 points when compared with control long COVID patients). Moreover, the effect sizes were medium to large. To our best knowledge, this is the first randomized controlled trial to find immediate and positive psychological effects of MBI in COVID patients with long-term effects. Indeed, although the effectiveness of MBI on stress, anxiety, and well-being management has been extensively studied in recent years (for recent reviews and meta-analysis see [32,33]), most studies were conducted during the pandemic (e.g., lockdown) or immediately after. To the question of whether persistent symptoms, particularly emotional and psychological disorders, can be treated by MBI, we can reasonably answer in the affirmative in light of the results reported herein. 

According to the *HADS*, *CFQ 11*, and *SSQ* scores, at baseline, all patients enrolled in this study reported anxiety and depressive symptomatology (scores on *HADS-A* and *HADS-D* above 8), elevated levels of mental and physical fatigue (*CFQ 11* Likert scoring = 29.3 ± 3.3 for G-Con and 28.2 ± 3.3 for G-Int), as well as impaired sleep quality (*SSQ* scores below 17). They also reported moderate muscular pain, joint pain, and headaches (*VAS* ratings of 4.5 to 7.4; [34]) as well as mood disturbances. By contrast, healthy individuals reported no anxiety or depressive symptomatology, no pain, no fatigue, as well as no sleep or mood disorders. Overall, our results are consistent with previous COVID-19 studies [12,35,36,37], with, however, some higher/lower values. For instance, in recovered COVID-19 patients (n = 67), Townsend et al. [36] reported a mean physical fatigue score of *CFQ 11_PHY_* = 14.5 ± 2.9 (vs. 18.1 ± 2.3 in our study) and a mental fatigue score of *CFQ 11_MEN_* = 5.5 ± 2.3 (vs. 10.6 ± 1.5). In another study that enrolled 458 non-hospitalized subjects 1.5 to 6 months after the acute phase, Stavem et al. [37] reported, using an online survey, a physical subscale score of *CFQ 11_PHY_* = 10.1 ± 3.8 and a mental subscale score of *CFQ 11_MEN_* = 5.0 ± 1.8. However, differences in population characteristics, methods (e.g., variations in scoring, online vs. in-person surveys), and timing relative to COVID onset make direct comparisons between studies difficult. In particular, significant sex differences in long COVID have been shown [38,39]. Female patients were at greater risk than male patients for long COVID syndrome and experienced greater fatigue, pain, anxiety, and depression [40]. As our study included almost twice as many women as the study led by [37], it is therefore not surprising to find higher scores on the *CFQ 11* questionnaire. 

Relative to cognitive functioning, the three main findings of the present study are that: (*i*) long COVID patients showed lower performances at baseline compared to a healthy population, (*ii*) when a performance improvement was manifest within long COVID groups it was systematically in favor of the intervention one (i.e., no evident cognitive improvement of the control group), and (*iii*) long-lasting effects of the MBI sessions could still be observed, and even amplified, 1 week after the neuro-meditation intervention.

Longer RTs and impaired performances could be observed at baseline in all of the cognitive tasks performed by long COVID patients of the present study, in comparison to a group of healthy participants. Depending on the task requirements, this observation manifested either through a reduced score (Corsi block-tapping task), a greater deviation from a target (psychomotor task), or via slowest response times. Specifically, to the *CRT*, *Simon*, and *PC* tasks, the slowing in information processing speed extended from ~50 (i.e., response inhibition task) to ~400 ms (i.e., pattern recognition task). Such increases make those RTs largely different from typical reports on these tasks, as illustrated by the normative group’s own standard deviations. Alarming relative to our participants’ condition, this finding, however, does not appear anecdotal as it is consistent with previous reports on long COVID patients for ~70% of which cognitive dysfunction is a symptom [3,41,42,43]. Neural mechanisms behind this impairment have been suggested to relate to a loss of gray matter notably concentrated in the left hemisphere, whose structural changes could turn into functional deficits and affect most, if not all, cognitive functions [44]. The present study provides further insight into the potential confounding factors behind COVID patient-related cognitive decline. Indeed, regarding the study timing, design, and the population tested, it can be argued that the overall cognitive deficit observed in COVID patients is not attributable to the general stress caused by the pandemic nor to a general response to acute respiratory distress, but rather constitutes a key feature of post-COVID-19 pathology.

Long COVID patients related cognitive alteration can be numerically observed for each of the tasks performed. The fact that it did not systematically reach the level of statistical significance could be explained by the heterogeneity of the severity of the participants’ symptoms [45]. In spite of this, findings consistent with the literature could be reported and specific observations could still be denoted that might represent the long COVID signature. For instance, while G-Nor typically showed faster RTs for CO than IN trials in the pattern comparison task at baseline, long COVID patients surprisingly revealed an opposite behavior. This phenomenon, representative of a cognitive control adjustment, typically occurs in situations of increased uncertainty: When a stimulus is likely to induce inappropriate behaviors, participants adopt a more conservative strategy to increase accuracy and prevent error [46]. With our participants, it is thus possible that the “brain fog” experienced by ~80% of the patients post-infection could have affected the a priori easiest, CO pattern recognition trials due to these trials’ lower dissimilarity. Facing these trials, the implementation of increased top-down processes could have ensured a steady accuracy yet at the expense of a slower response speed, thus explaining why other, faster tasks were not impacted [47]. In addition, it is noteworthy that long COVID patients showed reduced psychomotor performances as this assessment tool remains poorly used within batteries of cognitive assessment tasks. The fact that the pursuit rotor task was affected, yet to a lesser extent than pure cognitive tests, could be explained by the lower relative cognitive demand and higher motoric requirement in this type of performance [48]. Mechanistically, the above-mentioned loss of gray matter in long COVID conditions could originate from this particular impairment, as well as any extensive (yet downregulated) projections from the prefrontal cortex to motor areas [49]. Taken together, these results add to the sparse literature assessing the cognitive consequences of long COVID on patients and reporting a multifaceted cognitive deficit, e.g., memory, executive function and language [50], processing speed, attention, executive function and working memory [47], verbal reasoning, multistage planning and spatial attention [45], inattention, disorientation, and poorly organized movements [8]. 

The MBI proposed in the current protocol consisted of ten 30-min sessions of a Rebalance^®^ program spread over 3–4 weeks and induced a positive effect on most of the cognitive performances at Post1. Specifically, only G-Int showed a change in performance in the CRT with faster RTs between baseline and Post1 for a maintained accuracy. Similarly in the PC, it is remarkable that the difference with G-Nor’s RTs on CO trials was manifest at baseline for both the two long COVID groups, yet was not evident anymore at Post1 for the G-Int only, an observation suggesting pattern recognition capacities closer to a healthy population and further supported by the trend to improvement on IN trials’ RT at Post1 in G-Int only. In the same dynamic, only G-Int showed a trend to an improved selective inhibitory control over impulsive reactions in the Simon task at Post1. Within this framework, it appears that the MBI sessions incurred favorable outcomes on cognitive performances requiring information processing speed. In contrast to these positive effects, the memory task and pursuit rotor task did not show any benefits of the neuro-meditation program, but rather demonstrated steady performances for the two long COVID groups over the whole protocol duration. Notably, these two tasks differ from others in the fact that they do not stress the participant’s information processing speed to a similar extent: the Corsi task measures performance irrespective of any user’s time pressure while the target’s regular route of the psychomotor task does not induce any uncertainty. It can then be suggested that when long COVID patients are given the time to provide an accurate behavior, the intervention’s ergogenic effects vanish. Such phenomenon could be explained by ego depletion models of cognitive resources, stating that the higher the pool of cognitive resources, the greater the cognitive control performance in high-demanding situations [51]. Applied to our results, it is indeed possible that RT-based tasks exceeded G-Con resources at baseline and Post1, while G-Int progressively developed the ability to cope with this type of constraint from baseline to Post1 by leveling up their cognitive resources.

The MBI sessions not only induced an “immediate” positive effect on cognitive performance, but also induced delayed benefits, with even higher cognitive performances at Post2 than at Post1. The continuous improvement on the three RT-based cognitive tasks is made explicit through an even higher response speed for maintained accuracy and is noteworthy in so far as no MBI sessions were performed for one week. While this duration appears insufficient to be discussed in terms of benefits retention, it is still an encouraging observation for mental health maintenance. Mechanisms behind the immediate versus delayed improvements are likely plural and could overlap. It has recently been shown that the Rebalance^®^ program was associated with improved sympathetic control and autonomic regulation, lower blood pressure and resting heart rate, and increased heart rate variability [12]. All of these observations being known to relate to a reduced state of physiological stress, improved well-being, enhanced sleep quality, and increased self-regulatory capacity, it is reasonable to assume that the Rebalance^®^ program acted as a relief-based ergogenic solution against long COVID symptomatology, hence constituting a promising non-pharmacological approach to combating its cognitive debilitative signature.

Altogether, the subjective and objective measures of mental health reported in this study are consistent with each other. It has been recently discussed that the subjective experience of cognitive deficits in this population may be considered predictive of the need for further objective, cognitive assessment [52]. While the current protocol does not enable retroactively defining the appearance of the symptoms, it appears judicious to consider the scheduling of a Rebalance^®^ program as soon as subjective symptoms persist and prior to any cognitive decline objectivized. Indeed, from a chronological perspective, it has been observed that subjective manifestations generally succeed in neural changes and precede behavioral observations [3,41,42,43]. From this point of view, a practical implication of the current experiment would be to initiate a dose-response protocol for long COVID patients revealing early and/or altered signs of subjective alterations. In addition to improved sleep quality and state of mind, this could enable a reduction in daily risks that patients still have to face relating to time-constraining situations (e.g., moving, driving, socioprofessional behaviors). It would further align with the National Institute for Health and Care Excellence, according to which multidisciplinary models of care should target cognitive functioning among mental health and well-being.

Some limitations of our study should be mentioned. Firstly, the absence of a group carrying out a classical meditation program does not allow us to isolate the sole effects of the present neuromodulation practice. While future studies should strive to dissociate classical vs. digital intervention-related cognitive benefits, it is noteworthy to mention that [53] reported higher efficacy of mindfulness training from digital MBI through enhanced accessibility, standardization, and personalization of the training. Moreover, the fact that we conducted a second assessment (Post2) one week after the post-program session was relevant to test potential delayed effects (e.g., [16]), yet was not sufficient to conclude whether the observed benefits are persistent over time i.e., beyond 7 days. Therefore, future studies should test the maintenance of these effects in the medium and long term by carrying out, for instance, assessments 1, 3, and 6 months after the intervention period.

## 5. Conclusions

Neuro-meditation provided an effective, non-pharmacological treatment to combat subjective and cognitive impairment in long COVID patients. Compared to a control group, the participants undertaking ten 30-min sessions of the Rebalance^®^ program revealed improved physical and mental fatigue, muscle and joint pain, symptoms of depression and anxiety, mood disturbances, sleep quality, as well as faster response speed in cognitive tasks simulating time-pressure scenarios. This study adds to the sparse literature retracing the long-lasting signature of COVID symptomatology. It also reveals that MBIs could constitute a useful tool to combat these symptoms. Further research should investigate the mechanisms explaining the apparent neural adjustments following the Rebalance^®^ program, as well as the optimal dose of MBI sessions in regard to the severity of patients’ symptoms.

## Figures and Tables

**Figure 2:**
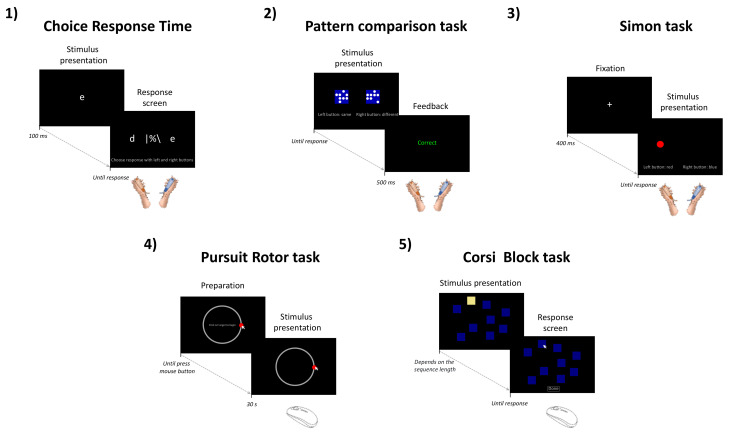
Illustration of the stimulus presentation sequence and timing for the five cognitive tasks used: (**1**) Choice response time, (**2**) pattern comparison task, (**3**) Simon task, (**4**) pursuit rotor task, and (**5**) Corsi block-tapping task.

**Table 1:** Main demographic and clinical characteristics of the study sample at baseline. Descriptive statistics are expressed as mean ± standard deviation (SD).

	Intervention Group G-Int (n = 17)	Control Group G-Con (n = 17)	Normative Control G-Nor (n = 15)
**Demographic characteristics**			
Age at the baseline visit (in years)	47.1 ± 8.3	48.7 ± 10.4	45.9 ± 11.8
Sex ratio (M/F)	0.3 (4/13)	0.4 (5/12)	0.5 (5/10)
ICU admission	2/17	1/17	0/15
**Vascular factors**			
Systolic blood pressure, mm Hg	123.8 ± 15.4	122.9 ± 11.8	124.6 ± 13.7
Diastolic blood pressure, mm Hg	75.8 ± 10.9	78 ± 9.7	73.3 ± 9.8
Heart rate at rest	72.6 ± 11.3	74.5 ± 16.1	67.5 ± 14.3
**Lifestyle factors**			
Severity of dyspnea (mMRC)	1.5 ± 0.9	1.4 ± 0.7	0.2 ± 0.4
Anxiety (HADS)	10.8 ± 3.5	12.9 ± 3.2	4.0 ± 2.4
Depression (HADS)	11.9 ± 2.7	12.5 ± 4.3	2.0 ± 1.3
Physical fatigue (CFQ)	17.8 ± 2.2	18.4 ± 2.4	6.8 ± 1.4
Mental fatigue (CFQ)	10.4 ± 1.6	10.9 ± 1.5	4 ± 0.4
Sleep quality (SSQ)	15.1 ± 3.8	14.0 ± 3.8	20.5 ± 3.1
Total Mood Disturbance (POMS-abbreviated)	45.5 ± 22.8	58.9 ± 22	−14.2 ± 11.3

ICU: Intensive Care Unit; mMRC: Modified Medical Research Council); HADS: Hospital Anxiety and Depression Scale; CFQ: Chalder Fatigue Scale; SSQ: Spiegel Sleep Questionnaire; POMS: Profile Of Mood States.

**Table 2:** Summary of the psychometric assessment results (questionnaires).

Questionnaire	Dimensions (Score Min–Max)	Group	Baseline	Post	Diff _Base-Post_
CFQ 11	Physical fatigue (0–21) ^†††^	G-Nor	6.8 ± 1.4	7.2 ± 1.1	+0.4
		G-Con	18.4 ± 2.4 ^∆∆∆^	17.8 ± 2.2 ^∆∆∆^	−0.6
		G-Int	17.8 ± 2.2 ^∆∆∆^	4.8 ± 4.1 °°°***	−13
	Mental fatigue (0–12) ^†††^	G-Nor	4 ± 0.4	4.3 ± 0.7	+0.3
		G-Con	10.9 ± 1.5 ^∆∆∆^	10.6 ± 1.7 ^∆∆∆^	−0.3
		G-Int	10.4 ± 1.6 ^∆∆∆^	2.7 ± 3.1 °°°***	−7.7
HADS	Anxiety (0–21) ^†^	G-Nor	4.0 ± 2.4	3.3 ± 1.2	−0.7
		G-Con	12.9 ± 3.2 ^∆∆∆^	11.4 ± 3.5 ^∆∆∆^	−1.5
		G-Int	10.8 ± 3.5 ^∆∆∆^	7.2 ± 3.0 ^∆∆^°°***	−3.6
	Depression (0–21) ^†††^	G-Nor	2.0 ± 1.3	2.7 ± 1.6	+0.7
		G-Con	12.5 ± 4.3 ^∆∆∆^	11.9 ± 4.6 ^∆∆∆^	−0.6
		G-Int	11.9 ± 2.7 ^∆∆∆^	6.7 ± 3.6 ^∆^°°°***	−5.2
mMRC	Dyspnea (0–4)	G-Nor	0.2 ± 0.4	0.2 ± 0.4	0
		G-Con	1.4 ± 0.7 ^∆∆∆^	1.2 ± 0.7 ^∆∆∆^	−0.2
		G-Int	1.5 ± 0.9 ^∆∆∆^	1.1 ± 0.7^∆∆^*	−0.4
VAS Pain	Muscle and Joint (0–10) ^†^	G-Nor	1.7 ± 1.5	1.0 ± 1.1	−0.7
		G-Con	7.1 ± 1.4 ^∆∆∆^	6.8 ± 1.4 ^∆∆∆^	−0.3
		G-Int	6.4 ± 1.6 ^∆∆∆^	4.6 ± 2.2 ^∆∆∆^°°°**	−1.8
	Headaches (0–10) ^††^	G-Nor	0.7 ± 1.0	0.3 ± 0.7	−0.4
		G-Con	7.2 ± 1.7 ^∆∆∆^	6.5 ± 2.2 ^∆∆∆^	−0.7
		G-Int	5.3 ± 2.6 ^∆∆∆^	2.8 ± 1.7 ^∆∆^°°°***	−2.5
SSQ	Sleep quality (0–30)	G-Nor	20.5 ± 3.1	22.1 ± 2.9	+1.6
		G-Con	14.0 ± 3.8 ^∆∆∆^	16.0 ± 3.1 ^∆∆∆^	+2.0
		G-Int	15.1 ± 3.8 ^∆∆∆^	18.5 ± 4.5 *	+3.4
POMS	Tension-Anxiety (0–24) ^†††^	G-Nor	4.8 ± 1.8	3.9 ± 2.6	−0.9
		G-Con	15.6 ± 4.1 ^∆∆∆^	15.6 ± 6.2 ^∆∆∆^	0
		G-Int	13.2 ± 5.2 ^∆∆∆^	7.5 ± 4.8 °°°***	−5.7
	Anger-Hostility (0–24) ^†††^	G-Nor	2.8 ± 1.7	2.0 ± 1.7	−0.8
		G-Con	11.9 ± 3.4 ^∆∆∆^	10.8 ± 6.1 ^∆∆∆^	−1.1
		G-Int	9.9 ± 5.9 ^∆∆∆^	4.5 ± 4.2 °°°***	−5.4
	Fatigue-Inertia (0–20) ^†††^	G-Nor	4.9 ± 3.5	3.3 ± 2.3	−1.6
		G-Con	16.4 ± 3.3 ^∆∆∆^	16.2 ± 3.4 ^∆∆∆^	−0.2
		G-Int	14.6 ± 2.8 ^∆∆∆^	9.1 ± 3.6 ^∆∆∆^°°°***	−5.5
	Depression-Dejection (0–28) ^††^	G-Nor	1.1 ± 1.5	0.5 ± 0.5	−0.6
		G-Con	14.0 ± 7.1 ^∆∆∆^	12.6 ± 8.2 ^∆∆∆^	−1.4
		G-Int	11.0 ± 6.4 ^∆∆∆^	5.8 ± 4.8 °°***	−5.2
	Esteem-related affect (0–24) ^†††^	G-Nor	18.4 ± 1.8	16.9 ± 2.8	−1.5
		G-Con	9.0 ± 3.3 ^∆∆∆^	9.5 ± 3.6 ^∆∆∆^	+0.5
		G-Int	11.1 ± 3.4 ^∆∆∆^	13.9 ± 3.2 °°**	+2.8
	Vigor-Activity (0–20)	G-Nor	14.5 ± 3.7	13.9 ± 3.5	−0.6
		G-Con	4.3 ± 3.3 ^∆∆∆^	4.2 ± 2.3 ^∆∆∆^	−0.1
		G-Int	5.8 ± 1.8 ^∆∆∆^	7.1 ± 4.1 ^∆∆∆^	+1.3
	Confusion-Bewilderment (0–24) ^†††^	G-Nor	4.9 ± 2.6	3.1 ± 2.2	−1.8
		G-Con	14.4 ± 3.1 ^∆∆∆^	14.2 ± 4.0 ^∆∆∆^	−0.2
		G-Int	13.7 ± 3.4 ^∆∆∆^	8.4 ± 3.2 ^∆∆∆^°°°***	−5.3
	Total Mood Disturbance (−44–76) ^†††^	G-Nor	−14.2 ± 11.3	−17.5 ± 14.0	−3.3
		G-Con	58.9 ± 22.0 ^∆∆∆^	55.6 ± 30.3 ^∆∆∆^	−3.3
		G-Int	45.5 ± 22.8 ^∆∆∆^	14.2 ± 21.7 ^∆∆^°°°***	−31.3

Notes: CFQ 11: Chalder Fatigue Scale; HADS: Hospital Anxiety and Depression Scale; mMRC: Modified medical research council dyspnea scale; VAS Pain: Visual Analog Scale for pain; SSQ: Spiegel Sleep Questionnaire; POMS: Profile Of Mood States. ^†^ Significant time–group interaction (*p* < 0.05) ^††^ Significant time–group interaction (*p* < 0.01) ^†††^ Significant time–group interaction (*p* < 0.001); ^∆^ Significantly different from G-Nor (*p* < 0.05) ^∆∆^ Significantly different from G-Nor (*p* < 0.01) ^∆∆∆^ Significantly different from G-Nor (*p* < 0.001); °° Significantly different from G-Con (*p* < 0.01) °°° Significantly different from G-Con (*p* < 0.001); * Significantly different from Baseline (*p* < 0.05) ** Significantly different from Baseline (*p* < 0.01) *** Significantly different from Baseline (*p* < 0.001).

**Table 3:** Cronbach’s alpha for the different self-administered questionnaires at both evaluation times (baseline, post).

Questionnaire	Subscale	Baseline	Post
CFQ 11	Physical fatigue	0.94	0.85
	Mental fatigue	0.93	0.90
HADS	Anxiety	0.88	0.81
	Depression	0.86	0.82
POMS	Tension	0.91	0.89
	Anger	0.92	0.88
	Fatigue-Inertia	0.94	0.92
	Depression	0.93	0.88
	Esteem-related affect	0.84	0.73
	Vigor	0.93	0.89
	Confusion-Bewilderment	0.86	0.79
SSQ		0.76	0.73

**Table 4:** Summary of cognitive task results.

Cognitive Task	Parameter	Group	Baseline	Post 1	Change _Post1-Base_ (%)	Post 2	Change _Post2-Post1_ (%)
CRT	Accuracy	G-Nor	0.98 ± 0.01	0.97 ± 0.03	−1.0		
		G-Con	0.95 ± 0.08	0.93 ± 0.1	−2.1		
		G-Int	0.96 ± 0.05	0.97 ± 0.05	+1.0	0.97 ± 0.04	0
	RT (ms) ^†^	G-Nor	491 ± 61	495 ± 67	+0.8		
		G-Con	587 ± 136	551 ± 121	−6.1		
		G-Int	593 ± 121	535 ± 83 **	−9.8	521 ± 86 ***	−2.6
Pattern comparison	Accuracy CO ^††^	G-Nor	0.97 ± 0.02	0.95 ± 0.04 *	−2.1		
		G-Con	0.98 ± 0.03	0.97 ± 0.03	−1.0		
		G-Int	0.98 ± 0.04	0.99 ± 0.03	+1.0	0.97 ± 0.04	−2.0
	RT CO (ms)	G-Nor	975 ± 121	949 ± 145	−2.7		
		G-Con	1371 ± 467 ^∆∆^	1353 ± 388 ^∆∆^	−1.3		
		G-Int	1277 ± 393 *^p^*^=0.06^	1198 ± 331	−6.2	1128 ± 281	−5.8
	Accuracy IN	G-Nor	0.94 ± 0.03	0.94 ± 0.03	0		
		G-Con	0.97 ± 0.03	0.96 ± 0.03	−1.0		
		G-Int	0.97 ± 0.03 ^∆∆^	0.98 ± 0.02 ^∆∆^	+1.0	0.96 ± 0.03	−2.0
	RT IN (ms) ^†^	G-Nor	1006 ± 130	974 ± 164	−3.2		
		G-Con	1277 ± 378	1275 ± 391	−0.2		
		G-Int	1244 ± 315	1156 ± 243	−7.1	1079 ± 213 ***	−6.7
Simon	Accuracy CO	G-Nor	0.98 ± 0.01	0.99 ± 0.01	+1.0		
		G-Con	1.0 ± 0.01 ^∆§^	1.0 ± 0.01 ^∆§^	0		
		G-Int	0.99 ± 0.01	0.99 ± 0.02	0	0.99 ± 0.01	0
	RT CON (ms)	G-Nor	448 ± 59	449 ± 64	+0.2		
		G-Con	515 ± 128	509 ± 131	−1.2		
		G-Int	511 ± 125	473 ± 88	−7.4	477 ± 134	+0.8
	Accuracy IN	G-Nor	0.95 ± 0.03	0.95 ± 0.04	0		
		G-Con	0.97 ± 0.03	0.96 ± 0.04	−1.0		
		G-Int	0.97 ± 0.03	0.97 ± 0.02	0	0.97 ± 0.03	0
	RT IN (ms)	G-Nor	479 ± 72	459 ± 66	−4.2		
		G-Con	532 ± 100	525 ± 99	−1.3		
		G-Int	539 ± 123	507 ± 86 *^p^*^=0.13^	−5.9	494 ± 134 **	−2.6
Psychomotor task	Time-on-target (%) ^†^	G-Nor	92.8 ± 1.8	90.3 ± 5.0	−2.7		
		G-Con	92.6 ± 2.6	93.9 ± 2.2^∆^	+1.4		
		G-Int	93.1 ± 2.9	93.4 ± 2.3	+0.3	93.3 ± 3.3	−0.1
	Mean deviation	G-Nor	14.7 ± 1.5	14.5 ± 1.7	−1.4		
		G-Con	18.4 ± 4.7 ^∆^	18.3 ± 4.8 ^∆^	−0.5		
		G-Int	17.2 ± 2.3	16.6 ± 2.5	−3.5	16.3 ± 2.3	−1.8
Corsi	Total score ^†††^	G-Nor	53.1 ± 8.6	65.0 ± 16.2 ***	+22.4		
		G-Con	44.5 ± 10.7 ^∆∆∆^	43.3 ± 12.8 ^∆∆^	−2.7		
		G-Int	43.5 ± 10.4 ^∆∆∆^	41.4 ± 9.9 ^∆∆^	−4.8	43.6 ± 10.4 ^∆∆∆^	+5.3
	Longest sequence ^†^	G-Nor	7.8 ± 0.7	8.2 ± 0.9 *^p^*^=0.09^	+5.1		
		G-Con	6.9 ± 1.3 ^∆∆^	7.0 ± 1.0 ^∆∆^	+1.4		
		G-Int	7.1 ± 0.9 ^∆∆^	6.9 ± 0.8 ^∆∆^	−2.8	7.0 ± 0.7 ^∆∆^	+1.4

Notes: CO: Congruent trials; IN: Incongruent trials; RT: Response time; ^†^ Significant time–group interaction (*p* < 0.05) ^††^ Significant time–group interaction (*p* < 0.01) ^†††^ Significant time–group interaction (*p* < 0.001); ^∆^ Significantly different from G-Nor (*p* < 0.05) ^∆∆^ Significantly different from G-Nor (*p* < 0.01) ^∆∆∆^ Significantly different from G-Nor (*p* < 0.001); ^§^ Significantly different from G-Int (*p* < 0.05); * Significantly different from Baseline (*p* < 0.05) ** Significantly different from Baseline (*p* < 0.01) *** Significantly different from Baseline (*p* < 0.001).

## Data Availability

Anonymized data supporting the study findings are available from the corresponding author upon reasonable request.
